# Editorial: Cognitive and Brain Plasticity Induced by Physical Exercise, Cognitive Training, Video Games, and Combined Interventions

**DOI:** 10.3389/fnhum.2018.00169

**Published:** 2018-05-07

**Authors:** Soledad Ballesteros, Claudia Voelcker-Rehage, Louis Bherer

**Affiliations:** ^1^Studies on Aging and Neurodegenerative Diseases Research Group, Universidad Nacional de Educación a Distancia (UNED), Madrid, Spain; ^2^Department of Basic Psychology II, Universidad Nacional de Educación a Distancia (UNED), Madrid, Spain; ^3^Institute of Human Movement Science and Health, Technische Universität Chemnitz, Chemnitz, Germany; ^4^Department of Medicine, Université de Montréal, Montreal, QC, Canada; ^5^Montreal Heart Institute, Montreal, QC, Canada; ^6^Institut Universitaire de Gériatrie de Montréal, Montreal, QC, Canada

**Keywords:** cognitive training, lifespan, multi-domain intervention, neuroplasticity, physical training, randomized controlled trial, video games, working memory

This Research Topic (RT) focused on recent research conducted in the field of cognitive and brain plasticity induced by physical activity, different types of cognitive training, including computerized interventions, learning therapy, video games, and combined intervention approaches as well as other forms of brain stimulation that target brain activity, including electroencephalography and neurofeedback. It contains 49 contributions to the topic, including original research articles (37), clinical trials (2), reviews (5), mini-reviews (2), hypothesis and theory (1), and corrections (2).

The premise of neuroplasticity on enhancing cognitive functioning among healthy as well as cognitively impaired individuals across the lifespan, and the potential of harnessing these processes to prevent cognitive decline attract substantial scientific and public interest. Indeed, the systematic evidence base for cognitive training, video games, physical exercise, and other forms of brain stimulation such as entrain brain activity is growing rapidly, thus paving the way for research geared at better understanding the underlying mechanisms and translation to clinical practice (Raz and Lindenberger, 2013). Studies in this field might contribute to improve our knowledge on cognitive and brain plasticity and be of great help for designing effective cognitive-enhancement interventions (see Karbach and Schubert, 2013). It is well-known that brain plasticity and its role in brain adaptation across the lifespan are influenced by other changes occurring as a result of environmental factors, personality variables and genetic and epigenetic factors (see Ballesteros et al., 2015). To date, most cognitive training studies have focused on measuring gains immediately after training, typically demonstrating effects on the trained tasks or closely-related transfer measures (i.e., near transfer). Yet the potency of cognitive training depends on evidence of: (1) far transfer from training to untrained functions; (2) the durability of training effects, including what booster regimens are needed to maintain cognitive benefits in young and older adults; and (3) the extent to which cognitive training can affect clinically meaningful outcomes.

Researchers are increasingly using cognitive training platforms and video games to investigate its impact on cognition and brain plasticity. Video game play is a very popular leisure activity. An interesting preliminary question is why consumers choose to download smartphones applications (apps) offering brain training. Torous et al. noticed that there is much interest in brain training apps among US younger people. Results from an online via internet survey with more than 3,000 participants suggest a high level of interest in these sort of programs. However, the data pointed out to the importance of expectations as both naïve participants as well as application-exposed participants showed a positive perception of brain training. These results suggest that people expect to improve with the use of brain-training apps. Importantly, brain training should focus on scientific research efficacy and generalizable benefits not in expectations of improvement (see Torous et al. correction).

Training with video games has shown to enhance moderately perceptual and cognitive functions in young and older individuals (for meta-analyses, see Lampit et al., 2014; Toril et al., 2014). Video games are inexpensive, gratifying and fun. Regular and occasional video game players reported significantly higher levels of well-being, but gaming could potentially lead to addiction, sedentary lifestyle and social isolation. Maximizing the benefits of video games will require studies dealing with questions such as: (1) what benefits should be expected from specific types of games; (2) how to account for individual differences; and (3) how to address expectancy bias and placebo effects in study designs. Physical activity has been repeatedly shown to improve cognitive functioning in all age groups, particularly in older adults. This Frontiers RT includes articles that investigate (1) the dose-response relationships of different types of exercise as well as the long-term effects of exercise on various cognitive domains, (2) the analysis of functional and structural as well as behavioral data, (3) the association between acute and chronic exercise effects, and (4) the potential of combining physical and cognitive exercise for enhancing cognitive performance and brain health.

In sum, the major aim of this RT is, therefore, to provide the interested reader with an objective picture of the state of the art on the influence of different types of interventions on cognition and brain state across the lifespan. Special emphasis is placed mainly in randomized controlled trials and longitudinal intervention studies conducted to assess possible transfer effects to cognitive and brain health in older adults.

## Cognitive training in young and older adults with traditional methods, computer-based activities, and video games

Several articles included in this Frontiers RT are longitudinal studies that have used different types of cognitive training programs to improve different aspects of cognition, including working memory, executive control and other cognitive functions in young and older adults. Other series of articles in this issue dealt with neurophysiological (ERPs) and neurofeedback (NFB) methods as a form of brain stimulation.

In their hypothesis and theory paper, Moreau et al. critically discuss pervasive statistical flaws in intervention designs for training-induced cognitive enhancement. That is (i) lack of power; (ii) sampling error; (iii) continuous variable splits; (iv) erroneous interpretations of correlated gain scores; (v) single transfer assessments; (vi) multiple comparisons; and (vii) publication bias. Similarly Cremen and Carson asked, “Have standard tests of cognitive function been misappropriated in the study of cognitive enhancement?” They argue that the latent constructs to which tests of cognitive functions relate are not usually subject to a sufficient level of analytic scrutiny. In addition they consider their neurophysiological correlates. Using linear mixed models, they found the efficacy of training and some maintenance effects. The individual characteristics considered contributed in some cases to explain the effects of training.

Lawton compared three intervention conditions in dyslexic second grade school children. Two of them target temporal dynamics of either the auditory or visual pathways, and the third condition consisted of a reading control group. The results pointed to the lack of synchronization of the activity of the magnocellular with the parvocellular visual pathway as the main cause of dyslexia, not the phonological deficits. Lawton proposed that visual movement direction-discrimination could be used as a tool for the successful treatment of this deficit Nouchi et al.

Nouchi et al. conducted a RCT to investigate whether the “Learning Therapy” (LT) improved a wide range of cognitive functions in older adults. They found benefits of LT on inhibition of executive functions. The article of Borella et al. explored in older adults whether age, education, vocabulary and baseline performance in a working memory task predict the short- and long-term gains and transfer effects of a verbal WM training. The authors selected four studies of the research group that adopted the verbal WM training procedure. Using linear mixed models, they found the efficacy of training and some maintenance effects. The individual characteristics considered contributed in some cases to explain the effects of training. Maraver et al. provided computerized training to groups of young adults to investigate the effects of training on WM or inhibitory control (IC) as compared to two-control groups, one passive and the other active, which performed non-executive control tasks. The trained groups improved in the trained task. More important were the pattern of near transfer effects as a function of the type of training. Only the IC group showed far transfer to reasoning (Raven test). Interestingly, these findings were obtained with just six training sessions. Schmicker et al. asked whether training young adults either attentional filtering or memory storage would influence decision-making assessed with the Iowa Gambling Task. All participants improved their performance in the trained task but decision-making was more influenced by training to filter out irrelevant information than by training to store items in WM. It seems that selective attention is more important for enhancing efficiency in decision-making.

Video games are perhaps the most popular computerized intervention approach to train different aspects of cognition. So far, the results of intervention studies are mixed with some studies reporting improvements after training in several aspects of cognition (e.g., Basak et al., 2008; Anguera et al., 2013; Ballesteros et al., 2014) while others have not found positive effects (e.g., Ackerman et al., 2010; Owen et al., 2010; Boot et al., 2013). Palaus et al. reviewed the relationship between the use of video games and their neural correlates. The final selection included 100 articles that provided functional data and 22 that measured structural brain changes. The authors established some links between the neural and cognitive aspects, including attention, cognitive control, visuospatial abilities, cognitive workload, and reward processing.

Toril et al. conducted an intervention study with experimental and control groups to investigate whether cognitively healthy older adults trained with non-action video games improve visuospatial working memory and episodic memory and whether these possible enhancements would persist 3 months after the end of training. The group trained with games showed post-training improvements in visuospatial working memory, and in short-term memory and episodic memory. Some results were maintained during a 3-month follow-up period. The authors concluded that older adults still retain some degree of plasticity and that video games seem to be an effective tool to improve some memory functions in aging. In contrast, the RCT conducted by Buitenweg et al. with healthy older adults assigned to a frequent or to an infrequent switching experimental condition, or to the active control group showed significant time effects on multiple transfer tasks in all three groups, probably as a result of expectancy and motivation. The authors concluded that the therapeutic value of using available training games to train the aging brain is modest. Possibly the use of different methods such as stimulating social interaction and training in groups by individual-adjusted variable exercises would produce better results.

Another type of training that becomes more popular involves the use of music or rhythm within a cognitive or video game setup. For example, rhythmic training has been successfully used to improve motor performance (e.g., gait) as well as cognitive and language skills. Begel et al. reviewed the games readily available in the market in order to see if it can be used for cognitive training in populations with motor or neurodevelopmental disorders (e.g., Parkinson's disease, ADHD). They concluded that none of the existing games provides sufficient temporal precision in stimulus presentation and/or data acquisition and that the available music games are not satisfying for implementing a rhythmic training protocol. The authors also provide guidelines for serious music games targeting rhythmic training in the future. The rational for rhythmic training is supported by some studies. An example is the study of Pollok et al. that used transcranial direct current stimulation to study potential superior synchronization in professional drummers compared to non-musician controls. Their data support the hypothesis that the posterior parietal cortex is involved in auditory-motor synchronization and extend previous findings by showing that its functional significance varies with musical expertise.

Effects of working memory training and training-related alterations in neural activity on dual-tasking in older adults were investigated by Heinzel et al. The training group participated in 12 sessions of an adaptive n-back training. At pre and post-measurement, a multimodal dual-task was performed in all participants to assess transfer effects. While no transfer to single-task performance was found, dual-task costs decreased at post measurement in the training, but not in the control group. Neural activity that changed in left dorsolateral prefrontal cortex (DLPFC) during one-back predicted post-training auditory dual-task costs, while neural activity changes in right DLPFC during three-back predicted visual dual-task costs. Results might indicate an improvement in central executive processing that could facilitate both working memory and dual-task coordination.

Teasdale et al. investigated whether individuals with MCI can benefit from a training program and improve their overall driving performance in a driving simulator. Therefore, older drivers with MCI participated in five training sessions in a simulator. They revealed gradual and significant decrease in the number of errors, indicating learning and safer driving and therewith the possibility to maintain driving skills and safe driving in MCI individuals. Another clinical population with which cognitive training is often used is patients that suffered from stroke, which often results in cognitive impairments in working memory, attention, and executive function. Van de Ven et al. conducted a systematic review of the evidence for computer-based cognitive training of executive dysfunctions after stroke. They reported that cognitive training could lead to improvement in tasks similar to the training (near transfer) and in tasks dissimilar to the training (far transfer). Studies evaluated neural effects and found changes in both functional and structural connectivity. The authors concluded that for most studies reporting positive findings, including neural changes, future research should address existing methodological limitations.

## Combined multi-domain interventions

Some studies looked at the effect of combining multi-domain intervention, such as different cognitive domains or cognitive intervention combined to fitness training regimes. For instance, Fraser et al. examined the dual-task benefits of combined physical and cognitive training in a sample of sedentary older adults, but failed to demonstrate a more beneficial effect of a combined physical and cognitive training as compared to physical and computer training combined with a respective control group (stretch and cognitive/computer training). Küper et al. used electrophysiology (ERP) to examine the effects of multi-domain cognitive training on performance in an untrained cue-based task switch paradigm featuring Stroop color words. Older adults were assigned to either a 4-month multi-domain cognitive training, a passive no-contact control group or an active (social) control group. Only the cognitive training group showed an increase in response accuracy at posttest, irrespective of task and trial type. Cognitive training was also associated with an overall increase in N2 amplitude and a decrease of P2 latency on single trials suggesting enhanced response selection and improved access to relevant stimulus-response mappings.

Recently, movement based video games (exergames) have been introduced to have the capability to improve cognitive function in older adults. During exergaming, participants are required to perform physical activities while being simultaneously surrounded by a cognitively challenging environment. Ordnung et al. investigated the effects of an exergame training over 6 weeks on cognitive, motor, and sensory functions in healthy old participants. However, gains in the trained exergames did not result in specific performance improvements.

## Neurofeedback (NFB) and electrophysiological studies

Neurofeedback (NFB) is becoming a popular method aimed at improving cognitive and behavioral performance and it is used as a treatment intervention. The goal of this type of intervention is to induce changes in the power of certain electroencephalography (EEG) bands to produce beneficial changes in cognition and motor activity. Rogala et al. reviewed the evidence that support the validity of several NFB protocols. The article highlights that the methodology used in most of the reviewed experiments did not enable proper targeting of the brain regions that control the desired cognitive changes and made a series of recommendations for improving the NFB training efficacy. Paluch et al. conducted an experiment using EEG-NFB with young adults and conclude that the activity from the EEG electrodes might be overwhelmed by the easier to control electro-miographyc signals. The authors advice the NFB community to develop, validate and implement efficient automatic artifact detection algorithms. Cowley et al. presented the results of a RCT intervention that used NFB therapy for adults with Attention Deficit/Hyperactivity Disorder (ADHD/ADD). Preliminary results suggest that NFB improved self-reported ADHD symptoms, but did not show transfer of leaning in a computerized attention test (T.O.V.A.). Hudak et al. reported an effect of NFB in reducing impulsive behavior via the strengthening of frontal lobe functioning. The randomized, controlled functional near-infrared spectroscopy (fNIRS) NFB intervention study tested its efficacy in an ADHD adult subgroup with high impulsivity. The reduction in the commission of errors on a no-go task, and an increase in prefrontal oxygenated hemoglobin concentration in the experimental group only, together with other findings suggest the potential of NFB in reducing impulsive behaviors by improving frontal lobe functioning. In addition, the authors argued that the use of virtual reality with NFB might improve the ecological validity of the training situation. This could affect positively transfer of the just acquired skills to real life.

Peters et al. investigated in an event–related potential (ERP) study conducted with young adults whether spatial frequency training modulates neural face perception. Interestingly, the authors showed that training effects based on a task using low-level stimuli, transfer to performance on a higher-level object-processing task. Interestingly, training the use of specific spatial frequency information was found to affect neural processing of facial information. These findings may have practical application to improve face recognition in people with atypical spatial frequency processing, including people suffering from cataracts or Autism Spectrum Disorder.

Gajewski et al. conducted a RCT to investigate the deficits in performance and EEG activity in workers with repetitive and unchallenging work. The results showed that the 3-month training protocol improved accuracy performance and affected the electrophysiological correlates of retrieval of stimulus-response sets (P2), response selection (N2), and error detection (Ne). Importantly, at the 3-month follow-up assessment most of the induced changes at the behavioral and EEG levels were maintained. It appears that cognitive training is useful for improving executive functions in workers with unchallenging work.

Wei et al. investigated whether structural differences in the hippocampus could explain sex difference in a 3D mental rotation task. Males had a larger anterior hippocampus and gray matter volume of the right anterior hippocampus was significantly correlated with performance in a 3D mental rotation task and could explain sex differences in mental rotation. Results suggest that the structural difference between males' and females' right anterior hippocampus was a neurobiological substrate for the sex difference in 3D mental rotation.

Hallock et al. presented the results of a meta-analysis that included 14 published studies that investigated the effects of cognitive training on measures of cognition and function measures in patients that have suffered traumatic brain injury (TBI). They showed a small but significant effect of cognitive training with no evidence of publication bias and a moderate effect size for overall functional outcomes and possible publication bias. In addition, the authors found significant effects only for executive function and verbal memory. The findings indicated that training is moderately effective in improving cognition and function in TBI patients.

## Fitness effects on cognition and brain anatomy

It is well-accepted that an active cognitive and physical lifestyle can reduce the risk of cognitive decline and dementia with aging (Valenzuela and Sachdev, 2006; Ngandu et al., 2015). Physical training promotes cognitive and functional brain plasticity in older adults (Nagamatsu et al., 2012; Kattenstroth et al., 2013). An important question is whether cognitive and physical training may increase white matter integrity (WMI), which is deteriorated in cognitive impaired patients. Fissler et al. did not find evidence that short-term cognitive or physical training were related to changes in WMI (hippocampus and prefrontal white matter tracks) in older adults at risk of dementia despite activity-related cognitive changes. However, the authors found positive associations between the two targeted training outcomes and WMI. This result opens the path for a potential of long-term activities to affect WMI. The article of Fletcher et al. tested the hypothesis that if cardiorespiratory fitness counteracts the negative effects of aging; the regions that show the greatest age-related volumetric loss should show the largest beneficial effects of physical exercise. The structural magnetic resonance imaging (MRI) data from 54 healthy elders showed that lower fitness and older age are associated with atrophy in different brain areas, but the profiles of age and fitness effects were not totally overlapping. While brain areas such as the precentral gyrus, the superior temporal sulcus, and some parts of the medial temporal lobe were affected by both fitness and aging, other areas (regions of the frontal, parietal, and temporal cortex) were only affected by aging while other brain regions in the basal ganglia were only affected by fitness. These results support the idea that aging and fitness have differential effects on the brain and leads to the conclusion that fitness couldn't revert all the negative effects of aging.

Wengaard et al. investigated the association of physical fitness, measured as maximal oxygen uptake (VO2max), muscle mass, weekly training, and cognitive function in the executive domains of selective attention and inhibitory control, in healthy male high-school students. Only maximal oxygen uptake was positively associated with cognitive function. Kleemeyer et al. addressed the theme of neural specificity (understood as the degree to which neural representations of different types of stimuli can be distinguished). Neural specificity declines with aging and its reduction is associated with lower cognitive performance (Park et al., 2010). The authors concluded that physical activity might protect against age-related declines in neural specificity. The study tested the hypothesis that exercise-induced improvements in fitness would be related to greater neural specificity in a group of 52 older adults randomly assigned to a high-intensity training group or to a low-intensity training group. The hypothesis was confirmed by the results of an fMRI experiment in which the participants were presented with pictures of faces and buildings. Participants whose physical fitness improved more also showed more changes in neural specificity. Hsu et al. conducted a 6-month RCT to investigate the impact of aerobic exercise (AE) training in older adults on fronto-parietal network connectivity. The results suggested that AT improve mobility in older adults with mild subcortical ischemic cognitive impairments (see Hsu et al. correction).

Rehfeld et al. compared the effects of an 18-month dancing intervention and traditional health fitness training on volumes of hippocampal subfields and balance abilities. Both, members of the dance-intervention and members of the fitness intervention revealed hippocampal volume increases mainly in the left hippocampus. The dancers showed additional increases in the left dentate gyrus and the right subiculum. Moreover, only the dancers achieved a significant increase in the balance composite score. Hence, dancing constitutes a promising candidate in counteracting the age-related decline in physical and mental abilities. Kandola et al. performed a review on how aerobic exercise is associated with cognitive enhancements and stimulates a cascade of neuroplastic mechanisms that support improvements in hippocampal functioning. Therefore, they summarized the animal and human literature. Using the examples of schizophrenia and major depressive disorder, they proposed the utility and implementation of an aerobic intervention to the clinical domain.

Panda et al. investigated how meditation alters the default mode network (DMN) using simultaneous EEG and functional MRI to compare the spatial extents and temporal dynamics of the DMN during rest and meditation. They found alterations in the duration of the DMN microstate in meditators highlighting the role of meditation practice in producing durable changes in temporal dynamics of the DMN. Su et al. also studied the effect of mindfulness training and its modulation on pain perception. They compare participants' brain-behavior response before and after a 6-week mindfulness-based stress reduction (MBSR) training course on mindfulness in relation to pain modulation using pain questionnaires and resting-state fMRI. They observed that the pain-afflicted group experienced significantly less pain after the mindfulness treatment than before, in conjunction with increased brain connectivity. These results suggest that mindfulness training can modulate the brain network dynamics underlying the subjective experience of pain. Cakmak et al. investigated the potential structural cortical plasticity in Sufi Whirling Dervishes, a form of physically active meditation. Results demonstrated significantly thinner cortical areas for Sufi Whirling Dervishes subjects compared with the control group in the DMN as well as in motion perception and discrimination areas of the brain.

Dordevic et al. conducted a feasibility study to assess the effect of 1-month of slackline-training on different components of balancing ability and its transfer effects on non-visual-dependent spatial orientation abilities. The training group performed significantly better on the closed-eyes conditions of the clinical balance test and in the vestibular condition of the orientation test, probably caused by a positive influence of slackline-training on the vestibular system function. Beck et al. investigated whether and how academic achievements in children can benefit from specific types of motor activities (e.g., fine and gross) integrated in to learning activities, here math lessons. They conducted a 6-week within school cluster-randomized intervention study. The study demonstrates that motor enriched learning activities can improve mathematical performance, particularly in normal math performers (but less in low math performers).

Godde and Volcker-Rehage conducted an intervention study to investigate whether a walking intervention and a motor control intervention reduce the cognitive brain resources that people recruited while performing motor tasks. Brain activation was assessed pre and post-intervention while the participants were imagining forward and backward walking. The results showed a positive association between initial motor status and activation decrease in the dorsolateral prefrontal cortex from pre-to-post assessment in both trained groups, suggesting that training effects might improve situations where people have to perform motor and cognitive tasks at the same time.

Condello et al. performed a cross-sectional study and investigated whether physical activity (PA) habits may positively impact performance of the orienting and executive control networks in community-dwelling older adults and diabetics, who are at risk of cognitive dysfunction. Results suggest that high PA levels exert beneficial, but differentiated effects on processing speed and attentional networks performance in aging individuals that partially counteract the detrimental effects of advancing age and diabetic status. Nadeau et al. assessed the impact of a 3-month aerobic exercise training using a stationary bicycle on a set of gait parameters and executive functions in sedentary Parkinson's disease (PD) patients and healthy controls. Aerobic capacity, as well as performance of motor learning and on cognitive inhibition, increased significantly in both groups after the training regimen, but only PD patients improved their walking speed and cadence. In PD patients, training-related improvements in aerobic capacity correlated positively with improvements in walking speed. Gait improvements seem to be specific to the type of motor activity practiced during exercise (i.e., pedaling), where improvements in cognitive inhibition were rather unspecific to the type of training (i.e., improvement of cardiovascular capacity).

Tan et al. investigated the cortical structural and functional differences in athletes and novices by comparing gray matter volumes and resting-state functional connectivity in 21 basketball players and 21 novices with MRI techniques. They found larger gray matter volume in basketball players than in novices in many regions (anterior insula, inferior frontal gyrus, inferior parietal lobule, and right anterior cingulate cortex). They also reported higher functional connectivity in the DMN, salience network and executive control network in basketball players compared to novices. Yu et al. investigated the effects of modified constraint induced movement therapy (CIMT) in acute subcortical cerebral infarction. The results showed positive effects immediatelly after treatment but long-term effects were not found.

In a very interesting review, Stillman et al. used a macroscopic lens to identify potential brain and behavioral/socioemotional mediators of the association between physical activity and cognitive function. They first summarized what is known regarding cellular and molecular mechanisms, and then discussed evidence for brain systems and behavioral/socioemotional pathways by which physical activity could impact cognition. The review proposes a number of potential moderator and mediators of the relationship between physical activity and cognitive performances. This review offers a theoretical context that could be useful to organize the current scientific knowledge regarding physical activity and brain structure and functions. It could also lead to a more complete characterization of the processes by which physical activity influences neurocognitive function, as well as a greater variety of targets for modifying neurocognitive function in clinical contexts.

Finally, some studies adopted the approach of studying acute exercise effects. For instance, Spring et al. investigation revealed that cardiovascular exercise could reduce movement related cortical potentials assessed with EEG while performing a knee extension task, which was related to muscle alterations and resulted in the inability to produce a maximal voluntary contraction.

Lundbye-Jensen et al. investigated whether acute exercise protocols following motor skill practice in a school setting can improve long-term retention of motor memory in preadolescent children and were able to show that acute intense intermittent exercise performed immediately after motor skill acquisition facilitates long-term motor memory in pre-adolescent children, presumably by promoting memory consolidation.

To summarize, the series of review and research articles that compose this Frontiers RT provide comprehensive information on the importance of different types of interventions as a way of enhancing some cognitive functions across the lifespan. We hope that this RT will prompt a critical “thinking” in the context of the scientific community on the possibilities of improving cognition and well-being, providing clues for conducting further research intervention studies in the next years. We also hope that the information included in this RT will move the scientific community to generate new research projects directed to overcome some shortcomings appearing in the field of neuroplasticity, promoting cognitive enhancement and improving a the quality of life of young and older adults.

## Author contributions

All authors listed have made a substantial, direct and intellectual contribution to the work, and approved it for publication.

### Conflict of interest statement

The authors declare that the research was conducted in the absence of any commercial or financial relationships that could be construed as a potential conflict of interest.
